# Adaptation of a simple dipstick test for detection of *Vibrio cholerae* O1 and O139 in environmental water

**DOI:** 10.3389/fmicb.2013.00320

**Published:** 2013-10-29

**Authors:** Subhra Chakraborty, Munirul Alam, Heather M. Scobie, David A. Sack

**Affiliations:** ^1^Department of International Health, Johns Hopkins Bloomberg School of Public HealthBaltimore, MD, USA; ^2^International Centre for Diarrhoeal Disease ResearchDhaka, Bangladesh

**Keywords:** cholera, dipstick, Bangladesh, environmental water, *Vibrio cholerae*

## Abstract

The presence of *Vibrio cholerae *in the environment is key to understanding the epidemiology of cholera. The gold standard for laboratory confirmation of *V. cholerae* from water is a culture method, but this requires laboratory infrastructure. A rapid diagnostic test that is simple, inexpensive, and can be deployed widely would be useful for confirming *V. cholerae* in samples of environmental water. Here, we evaluated a dipstick test to detect *V. cholerae* O1 and O139 from environmental water samples in spiked samples and under field conditions. When environmental water samples were incubated in alkaline peptone water for 24 h at room temperature, samples spiked with <10 CFU could be detected using the dipstick test. When compared to culture, the test was 89% sensitive and 100% specific with environmental samples.

## INTRODUCTION

Cholera is transmitted through water contaminated with *Vibrio cholerae*. Methods for identifying culturable *V. cholerae *from water and sewage include culture and PCR techniques (Barrett et al., 1980; Fields et al., 1992; Alam et al., 2010), but efforts for detecting *V. cholerae* in environmental samples have previously been limited by laboratory testing capacity in areas where cholera most often occurs and the difficulty of achieving reliable results in these areas.

Recently a dipstick rapid test, based on detection of LPS antigen was introduced for detecting *V. cholerae* directly from fecal samples (Crystal VC kit, Span Diagnostics Ltd., Udhna, Surat, India). When watery fecal samples were tested directly, using culture method as gold standard the test was found to be sensitive (>90%) and moderately specific (~70%; Ingram et al., 1996; Rabbani et al., 2001; Bhuiyan et al., 2003; Nato et al., 2003; Wang et al., 2006; Harris et al., 2009; Mukherjee et al., 2010; Rosewell et al., 2012; Sinha et al., 2012; Boncy et al., 2013). The test can also be carried out with rectal swab specimens if these samples are first incubated in alkaline peptone water (APW) for 4 to 6 h, a vibrio-selective enrichment step, prior to testing with the dipstick (Bhuiyan et al., 2003). We felt that this dipstick test might be adapted for use in detecting *V. cholerae *O1 and O139 from environmental water samples in areas at risk for cholera if the specimen was incubated in APW first in order to amplify the concentration of vibrios in the specimen.

## MATERIALS AND METHODS

First, we carried out dipstick experiments with water samples from the Chesapeake Bay that were spiked with 10-fold serial dilutions of *V. cholerae* serotype O1 or O139. We placed 1 mL of the spiked water samples into 9 mL of APW, followed by incubation at temperatures of 22, 30, and 37°C (**Table 1**). The concentration of bacteria added to spike the water was determined by plating serial dilutions of the initial inoculum. We tested the APW after 2, 4, 6 and 24 h with the dipstick using methods as described by the manufacturer for fecal samples.

**Table 1:** Dipstick detection of *Vibrio cholerae*-containing water samples incubated in alkaline peptone water (APW).

*V. cholerae *serogroup O1			*V. cholerae *serogroup O139		
CFU/1 mL water added to APW	Incubation time/temperature		CFU/1 mL water added to APW	Incubation time/temperature	
	6 h/37°C	24 h/22°C		6 h/37°C	24 h/22°C
2000	+	+	1800	+	+
200	+	+	180	+	+
20	-	+	18	-	+
2	-	+	2	-	+
0	-	-	0	-	-

*Crystal VC test was read as positive (+) if *V. cholerae *O1 or O139 and control bands were visible, or negative (-) if only the control band was visible, according to manufacturer’s instructions.
*

Next, we applied the dipstick testing method to environmental water samples from Bangladesh. The samples included specimens from both urban and rural sites that were being collected as part of cholera epidemiology studies at the International Centre for Diarrheal Disease Research, Bangladesh (icddr,b) during March 2011–June 2012. Use of these samples allowed for a direct comparison of the dipstick testing method to bacterial culture methods used at icddr,b.

A total of 550 environmental water samples (200 mL each) were filtered through individual 0.22 micron polycarbonate membrane filters. The filters were then placed in 10 mL of phosphate buffered saline (PBS). After resuspension, 2 mL of the PBS solution was added to 18 mL of APW, followed by incubation for 24 h at room temperature (~22°C). The APW broth was then tested using the dipstick. The same APW was streaked onto taurocholate tellurite gelatin agar (TTGA) and thiosulfate-citrate-bile salts-sucrose (TCBS) plates, and colonies suspected as being *V. cholerae *from either plate were identified as *V. cholerae* O1 or O139 using standard methods. The results from the dipstick and cultures were recorded without knowledge of results from the other test.

## RESULTS

All spiked specimens were negative at 2 and 4 h, and only the APW spiked with greater than ~200 CFU were positive after 6 h of incubation at 37°C. However, after 24 h, all specimens were positive, including those spiked with <10 CFU and incubated at 30°C or at room temperature (22°C). The APW inoculated with unspiked water was consistently negative at all time points and temperatures (**Table 1**).

A positive dipstick reading occurred when the concentration of *V. cholerae *exceeded ~10^7^ per mL; thus, the small number of bacteria in the water that were added to the APW had to grow to this density to be detectable.

Among the 550 environmental samples tested in Bangladesh, 55 were positive for *V. cholerae* serotype O1 by the bacterial culture method, and 48 of these were also positive with the dipstick test (89% sensitivity). Of the 495 samples that were negative by culture, none were positive with the dipstick test (100% specificity). None of the samples were positive for *V. cholerae* O139 by dipstick or culture.

## DISCUSSION

This dipstick method following incubation in APW provides a simple procedure for detecting *V. cholerae* O1 and O139 from environmental water. Although the bacterial culture method did detect more positive specimens than the dipstick test, the dipstick test was still very sensitive. It should be noted that the culture methods used at the icddr,b included streaking onto both TCBS and TTGA agars. Incubation on these two media will generally be more sensitive than methods which use only one type of agar, as is carried out in most laboratories (Alam et al., 2010). Thus, the sensitivity of the dipstick method may be as sensitive as using culture with TCBS alone.

While our preliminary study used samples spiked with O139 as well as O1 serotypes, there were no O139 strains detected with the environmental samples from Bangladesh. It seems plausible that this method can be used for the detection of serotype O139 from environmental water, but this has not yet been validated in the field. Recently, there have been no clinical cases of cholera nor environmental isolations related to *V. cholerae* O139 in Bangladesh. The finding that there were no positive dipstick tests for O139 was reassuring.

The high specificity of the dipstick test (100%) in this evaluation differs from the results observed in clinical studies of fecal samples in which many dip stick positive tests could not be confirmed by culture suggesting a high rate of false positives (Rabbani et al., 2001; Harris et al., 2009; Rosewell et al., 2012). We believe that this difference is because we carried out the test on water samples after incubation in APW rather than directly testing as done in fecal specimens. The reason that some stool samples yield false positive results is not known, but by using an APW selective enrichment step, cross reacting substances are diluted and vibrio antigens are amplified. Our results confirm that APW enrichment of samples helps to improve the specificity of the test, as previously demonstrated (Wang et al., 2006). For water samples, incubation in APW allows growth of vibrio species to concentrations detectable with the dipstick.

This study used the Crystal VC dipstick to detect *V cholerae* as this has been the test most widely used in developing country settings. Other rapid tests for *V. cholerae* are also available (Qadri et al., 1995) and might also be useful since this method primarily relies on the ability of vibrios to grow and for the LPS signal to be amplified in the APW broth prior to testing.

Some limitations to this dipstick method should be mentioned. *V. cholerae* can exist as viable but non-culturable (VBNC) bacteria (Alam et al., 2007). The dipstick test relies on the ability of the bacteria to grow in the APW medium; thus, it is not able to detect VBNC vibrios. Secondly, the Crystal VC dipstick test detects the LPS of *V. cholerae *but does not differentiate toxigenic from non-toxigenic strains. Non-toxigenic strains of serogroups O1 and O139 may also occur in environmental water but do not carry the same epidemic implications (Faruque et al., 2004). Thus, it would seem that some positive samples should be validated with follow up cultures to determine if they are toxigenic and to more fully characterize the strains. However, it should be noted that standard culture methods also do not determine if the vibrio strains isolated produce toxin. Depending on the epidemiological circumstances, the finding of *V. cholerae* O1 and O139 in water samples should stimulate a public health response and it is imperative that the finding of a positive dip stick test is rapidly confirmed.

Another limitation of this method, as it was carried out in Bangladesh, included filtration through a 0.22 micron filter. For some cholera endemic areas, this method of filtration may not be possible. Thus, we are evaluating a less complex filtration method through cotton gauze, similar to the method used by Spira (Spira and Ahmed, 1981). Though filtration through gauze is likely to be somewhat less sensitive, it may still be useful in monitoring water samples. In fact the sensitivity of any assay also depends on the volume of water assayed, and no single test will be 100% sensitive.

We conclude that this dipstick method for testing environmental water samples is sensitive and specific for *V. cholerae *O1 and should be useful for water monitoring in cholera-endemic settings and in areas at risk for cholera. The method is technically simple and does not require use of an incubator. It is not “rapid” since an overnight incubation step is necessary but is “rapid” compared to traditional culture method.

## Conflict of Interest Statement

The authors declare that the research was conducted in the absence of any commercial or financial relationships that could be construed as a potential conflict of interest.
