# Erratum: Adaptation of a simple dipstick test for detection of *Vibrio cholerae* O1 and O139 in environmental water

**DOI:** 10.3389/fmicb.2013.00405

**Published:** 2013-12-24

**Authors:** Subhra Chakraborty, Munirul Alam, Heather M. Scobie, David A. Sack

**Affiliations:** ^1^Department of International Health, Johns Hopkins Bloomberg School of Public HealthBaltimore, MD, USA; ^2^Centre for Communicable Diseases, International Centre for Diarrhoeal Disease ResearchDhaka, Bangladesh

**Keywords:** cholera, dipstick, Bangladesh, environmental water, *Vibrio cholerae*

Two references cited in this article were incorrect. The reference to Ingram et al. (1996) should instead be Page et al. (2012) and the reference for Rabbani et al. (2001) should instead be Ley et al. (2012).
